# Supplementary material for: Combined Transcriptomic and Metabolomic Approach Revealed a Relationship between Light Control, Photoprotective Pigments, and Lipid Biosynthesis in Olives
Source: Int J Mol Sci. 2023 Sep 22;24(19):14448. doi: 10.3390/ijms241914448 (PMC10572622; doi:10.3390/ijms241914448)
Supplement: Supplementary file 1 [file ijms-24-14448-s001.zip › Supplementary_data.pdf]

Supplementary information

Combined Transcriptomic and Metabolomic Approach Revealed a Relationship between Light Control, Photoprotective Pigments, and Lipid Biosynthesis in Olives

Tiziana Maria Sirangelo [1], Ivano Forgione [1], Samanta Zelasco [1], Cinzia Benincasa [1], Enzo Perri [1], Elisa Vendramin [2], Federica Angilè [1,3], Francesco Paolo Fanizzi [3], Francesco Sunseri [4], Amelia Salimonti [1] and Fabrizio Carbone [1,*]

[1] Research Centre for Olive, Fruit and Citrus Crops, Council for Agricultural Research and Economics (CREA), Via Settimio Severo, 83, 87036 Rende, Italy; tizianasirangelo@gmail.com (T.M.S.);
ivano.forgione@crea.gov.it (I.F.); samanta.zelasco@crea.gov.it (S.Z.); cinzia.benincasa@crea.gov.it (C.B.); enzo.perri@crea.gov.it (E.P.); federica.angile@unisalento.it (F.A.); amelia.salimonti@crea.gov.it (A.S.)

[2] Research Centre for Olive, Fruit and Citrus Crops, Council for Agricultural Research and Economics (CREA), Via di Fioranello, 52, 00134 Rome, Italy; elisa.vendramin@crea.gov.it

[3] Department of Biological and Environmental Sciences and Technologies, University of Salento, Via Lecce-Monteroni, 73100 Lecce, Italy; fp.fanizzi@unisalento.it

[4] Department Agraria, University Mediterranea of Reggio Calabria, Località Feo di Vito, 89124 Reggio Calabria, Italy; francesco.sunseri@unirc.it

* Correspondence: fabrizio.carbone@crea.gov.it

Tables (Supplementary Tables.xlsx)

Table S1. Primers panel coordinates. The original target transcript code from Farga (Version 6, Cruz et al., 2016), the 5’ end and the 3’ end position of the left and right primers, together with the full amplified fragment ranges, are reported.

Table S2. Summary of reads filtering and alignment processes. Analysis performed on fruit samples collected during 2019 olive oil season.

Table S3. Summary of key DEGs during fruit ripening in each genotype grouped according to the principal functions included in the gene panel and to Venn groups (Fig. 2A). FC: Fold change; FDR: adjusted p-value.

Table S4. Summary of key DEGs in 'Cellina di Nardò' vs. 'Salella' comparison grouped according to the principal functions included in the gene panel and to Venn groups (Fig. 2B). FC: Fold change; FDR: adjusted p-value.

Table S5. Summary of anthocyanin contents in 'Cellina di Nardò', 'Ruveia' and 'Salella' olive extracts during ripening stages. Data for anthocyanin contents are reported as mg/L of cyanidin 3-ritinoside. The analyses were performed as nine replicas and the standard error of the mean (s.e.m.) is reported. Different letters indicate significant differences among mean values of the three cultivars. Asterisks indicate significant differences among mean values of the ripening stages for each cultivar.

Table S6. Summary of key DEGs in 'Ruveia' vs. 'Salella' comparison grouped according to the principal functions included in the gene panel and to Venn groups (Fig. 2C). FC: Fold change; FDR: adjusted p-value.

Table S7. Correlation analysis according to the threshold of Pearson’s correlation coefficient (PCC) > |0.90| among 18 Differential Accumulated Metabolites (DAMs) and 90 significant DEGs (Table 4 CdN *vs.* S comparison).

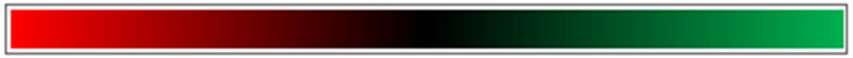

- 0.90 + 0.90

Table S8. Correlation analysis according to the threshold of PCC > |0.90| between 10 DAMs and 93 significant DEGs (Table 5 Ru *vs.* S comparison)

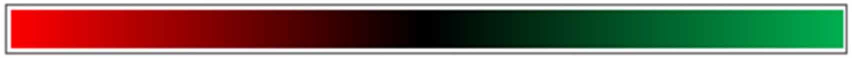

- 0.90 + 0.90

Table S9. HPLC-MS/MS analysis parameters: equation for external calibration curve, correlation coefficient R^2, molecular ion $[M\text{-}H]^{-1}$ monitored on the first quadrupole and major fragments monitored on the third quadrupole for each phenolic compound analysed by LC-MS/MS.

FIGURES

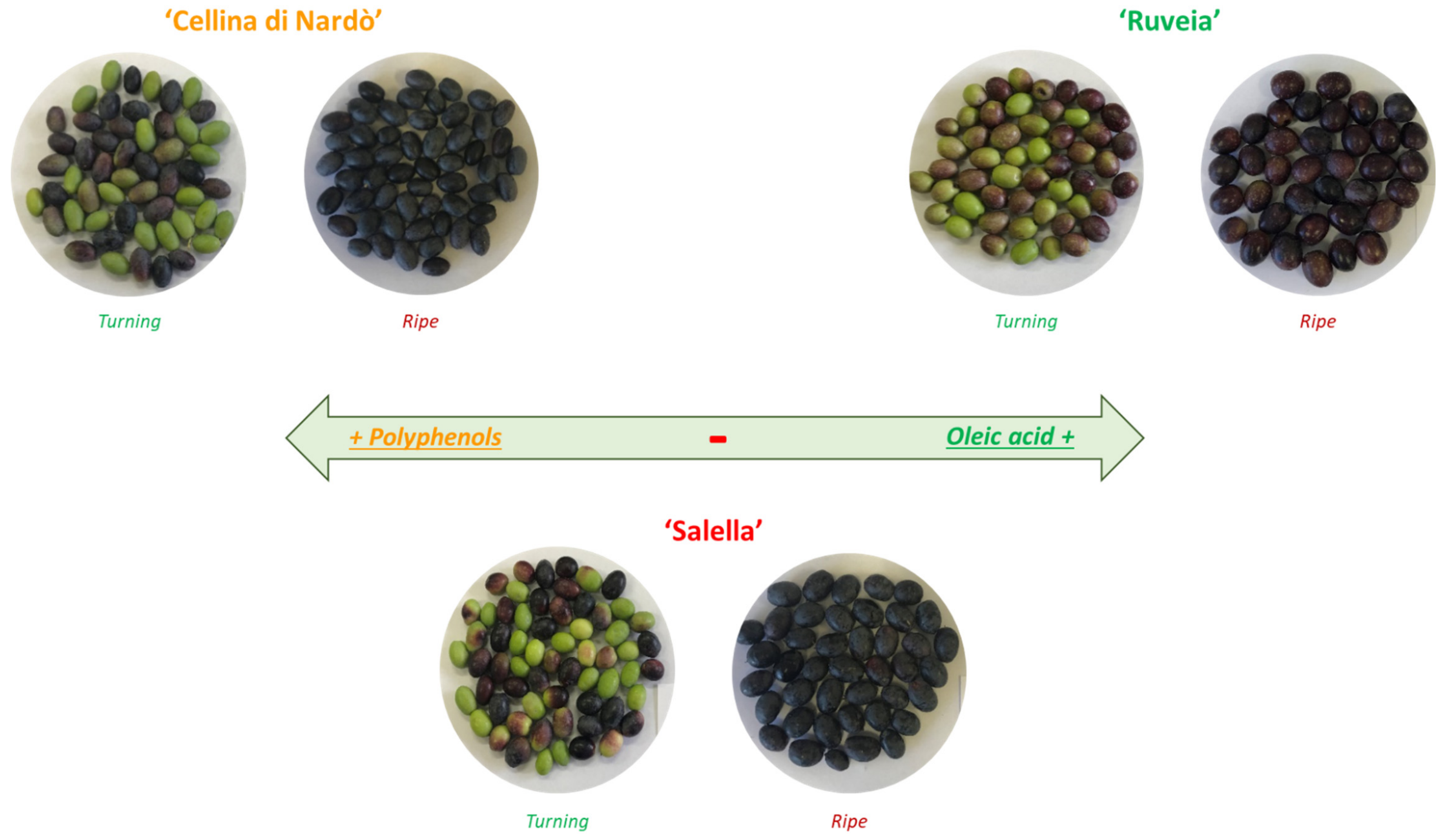

Figure S1. Ripening drupes (n = 50) of each cultivar, 'Cellina di Nardò' higher phenolic and aldehyde compounds of oleuropein and ligustroside (polyphenols) contents, 'Ruveia' higher oleic acid content, 'Salella' lower content of both chemical families, were randomly collected from three different 20-years-old trees, at turning (Jaén index between 2.0 and 2.5), and ripe stages (11 days after Turning).
